# Identifying risk factors for metastasis to the level VII lymph node in papillary thyroid carcinoma patients

**DOI:** 10.1186/s12893-020-0675-5

**Published:** 2020-01-14

**Authors:** Jian Chen, Deguang Zhang, Liang Fang, Gaofei He, Li Gao

**Affiliations:** Department of Head and Neck Surgery, Sir Run Run Shaw Hospital, Zhejiang University School of Medicine, 3 East Qingchun Road, Hangzhou, ZheJiang People’s Republic of China

**Keywords:** Papillary thyroid carcinoma, Level VII, Video-assisted approach, Lymph node dissection

## Abstract

**Background:**

The level VI lymph nodes are anatomically connected to the level VII lymph nodes and papillary thyroid carcinoma (PTC) can metastasis to both regions. The aim of this study was to identify clinicopathologic factors associated with level VII lymph node metastasis.

**Methods:**

Between March 2015 and September 2016, a total of 275 consecutive patients were operated on for PTC with prophylactic level VII dissection at the Sir Run Run Shaw Hospital, Zhejiang University School of Medicine, ZheJiang, China. Patients with PTC were performed via video-assisted approach. Multivariate logistic regression analysis was performed to evaluate the associations between clinicopathologic factors.

**Results:**

Of the 275 subjects enrolled in this study, 79 patients (28.73%) showed lymph node metastasis to the level VII. The multivariate logistic regression analysis showed ultrasonography-positive lymph nodes (*p* < 0.001), the location of primary carcinoma (*p* = 0.002) and hashimoto thyroiditis (HT) (*p* = 0.04) were associated with level VII lymph node metastasis.

**Conclusions:**

Based on the results of our study, we considered central-compartment lymph node dissection (CLND) as an integral strategy. On the basis of surgery safety, transcervical level VII lymph node dissection could be considered for PTC patients with high risk factors such as ultrasonography-positive lymph nodes, tumor located in middle and lower thirds of the thyroid lobe and the patients without HT. In future, prognostic significance of level VII lymph node dissection should be evaluated through long-term surveillance.

## Background

Papillary thyroid carcinoma (PTC) is the most common thyroid malignancy, accounting for 85% of all thyroid carcinomas [1]. Overall survival is considered excellent for most patients with PTC, exceeding 90% at 10 years [1, 2]. Lymph node dissection is critical in treating PTC, as these patients have high incidence of cervical lymph node metastasis (20~90%) [1, 3].

Central compartment lymph node metastasis is the most common due to its proximity to the thyroid. Prophylactic central-compartment lymph node dissection (CLND) is important for disease staging, determining doses for radioactive iodine, and eliminating a source of recurrent disease. Given reasons of all above, the prophylactic CLND remains a strategy to treat PTC. Ours is now performing routine prophylactic CLND.

Although according to the ATA guidelines, only high-risk cases require central lymphadenectomy. However, in recent years, a large number of clinical studies have found that the central lymph node metastasis rate of papillary thyroid carcinoma is higher. At the same time, CN_0_ papillary thyroid carcinoma patients with prophylactic central lymph node dissection can significantly improve the patient’s postoperative disease-free survival time and reduce the risk of recurrence. The Chinese Thyroid Association (CTA) guidelines also recommend that patients with papillary thyroid carcinoma routinely undergo a central lymph node dissection to reduce the risk of long-term recurrence.

Although it is clear that CLND remains an integral strategy, whether this should include the level VII lymph node is not clear. International guidelines used in PTC management inconsistently describe the inferior extent of CLND with sternal notch or the innominate vessel. The American Thyroid Association (ATA) management guidelines on PTC specify a CLND to target the level VI [1]. However, the ATA’s consensus statement on terminology of CLND differentially defines the innominate artery as the lower limit of a CLND [4]. This equates CLND to both level VI and level VII dissection. Additionally, the American Joint Committee on Cancer (AJCC) 7th edition/TMN Classification for Differentiated Thyroid Cancer originally considered level VII lymph nodes to be regional nodes, carrying the same prognosis as lateral-compartment lymph nodes (N1b); whereas the new AJCC 8th edition considered level VII lymph node metastases as central-compartment lymph nodes (N1a).

In this study, we aimed to investigate the relevance of various clinicopathologic factors as indicators of level VII lymph node metastasis.

## Methods

### Patients

Between March 2015 and September 2016, a total of 275 consecutive patients were operated on for PTC with prophylactic level VII dissection at the Sir Run Run Shaw Hospital, Zhejiang University School of Medicine, ZheJiang, China. The patients enrolled in this study met following criteria: 1) pathological confirmed PTC; 2) no previous thyroid surgery; 3) clinical-negative level VII lymph node which was defined as no suspicious metastatic lymph nodes on preoperative imaging studies and characteristics of gross metastatic lymph node. Selected patients were categorized into two groups according to whether or not they had pathological level VII lymph node metastasis. All patients provided written informed consent for their information to be stored in the hospital database and used for study, and this study was reviewed and approved by the Ethical Committee.

### Preoperative evaluations

All patients underwent a systematic evaluation including the surgeon and Multiple Disciplinary Team (MDT). Baseline laboratory evaluation included routine blood test, biochemistry test, thyroid function, parathyroid hormone (PTH) level, chest X-ray, electrocardiogram (ECG) and ultrasound (US). Patients with lymphadenopathy in the central or lateral neck, as detected by palpation or US, underwent a computed tomography (CT) scan of the neck and superior mediastinum as well as US-guided fine-needle aspiration (FNA) of the suspected lymph node. The mobility of the vocal fold was examined for all patients preoperatively.

### Surgical approaches

**Fig. 1:**
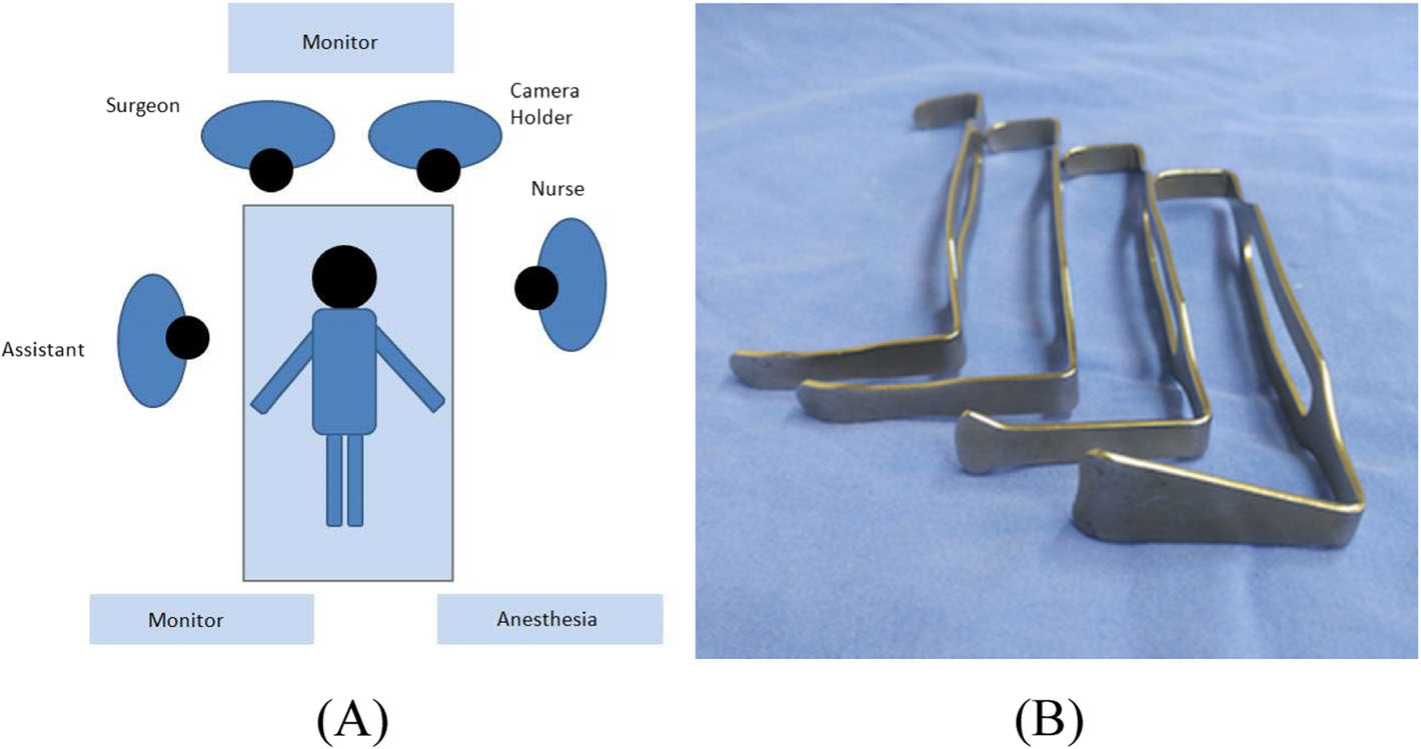
Position of surgeons and the surgical equipment for video-assisted level VII dissection. a Craniocaudal position of surgeons for level VII view. b Special self-developed retractors used for level VII lymph node dissection

**Fig. 2:**
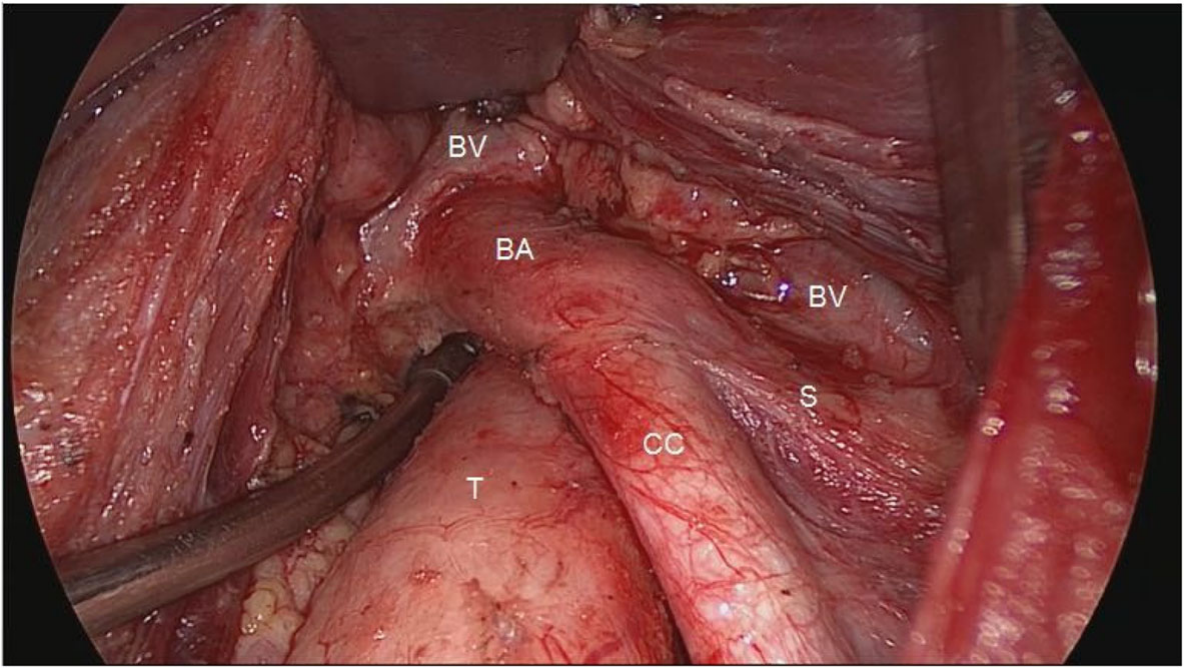
The surgical field after level VII lymph node dissection via transcervical video-assisted approach and the working space were maintained by special self-developed retractors. BA, brachiocephalic artery; BV, brachiocephalic vein; CC, common carotid artery; S, subclavian artery; T, trachea

The surgery was performed via video-assisted approach. Following routine thyroidectomy and CLND (level VI), prophylactic level VII dissection was performed via transcervical approach for all 275 patients. Specifically, the surgeon is positioned at the head of the patient to obtain a craniocaudal view of the level VII. The special self-developed thymus retractors were used to maintain the working space underneath the thymus (see Fig. 1). Level VI and level VII were divided at the level of the sternal notch intraoperatively and submitted separately for histopathological examination (see Fig. 2).

Vascularized parathyroid glands are generally preserved in situ. However, in instances of poor vascularization, pathologically-confirmed parathyroid tissue distinct from metastatic carcinoma was autotransplanted in brachioradialis or sternocleidomastoid muscle. The thymus was preserved in situ in cases with ectopic parathyroid glands.

### Postoperative care and follow-up

In the immediate postoperative period, all patients underwent thyroid stimulating hormone (TSH) suppression therapy using levothyroxine and monitoring of and serum intact PTH levels, serum ionized calcium, phosphorus, and magnesium. Clinical surveillance for recurrent disease included history and physical examination, thyroid function, serum thyroglobulin, and ultrasound. Radioiodine, CT imaging, and fine-needle aspiration cytology was performed on as indicated basis. The follow-up data were also entered into the database for analysis.

### Statistical analysis

Statistical analysis was performed using SPSS ver.20 (SPSS, Chicago, IL). The data are presented as means ± SDs for normally distributed variables. Comparisons of differences were made with a Student’s t test or Pearson’s chi-square as appropriate. The odds ratio (OR) and the 95% confidence interval (CI) for relationships between each variable and level VII metastasis were calculated using binary logistic regression. A *P* < 0.05 was considered statistically significant.

## Results

### Patient characteristics

The baseline characteristics of this study are summarized in Table 1. Of the 275 patients enrolled in this study, the age ranged from 20 to 78 years (mean 46.07 ± 12.03 year). The size of primary tumor ranged from 0.15 to 4.5 cm (mean 1.06 ± 0.75 cm). Two hundred twelve patients were women (77.09%), and 63 were men (22.91%). Overall, 275 patients with PTC who had an initial operation involving prophylactic video-assisted transcervical level VII dissection, 79 patients (28.73%) showed lymph node metastasis to the level VII. Based on US and frozen sections (FS) results, three major different types of surgical procedures were performed: 1) lobectomy with ipsilateral CLND (135 patients); 2) total thyroidectomy (TT) with ipsilateral/bilateral CLND (54 patients, 24 patients with ipsilateral CLND and 30 patients with bilateral CLND); 3) TT with CLND and ipsilateral/bilateral lateral-compartment lymph node dissection (LLND) (77 patients, 69 patients with ipsilateral LLND and 8 patients with bilateral LLND). Nine of two hundred seventy-five patients underwent lobectomy with ipsilateral CLND and LLND according to the decision of individual patients.

**Table 1:** Patient characteristics

Variable^a^	Total
	(*n* = 275)
Gender, n (%)	275 (100)
Male	63 (22.91)
Female	212 (77.09)
Age in years, mean (SD)	46.07 (12.03)
Size in cm, mean (SD)	1.06 (0.75)
Level VII lymph node metastasis, n (%)	
Yes	79 (28.73)
No	196 (71.27)
Produre, n (%)	
Lobectomy + CLND	135 (49.09)
TT + CLND	54 (19.64)
TT + CLND + LLND	77 (28.0)
Lobectomy + CLND + LLND	9 (3.27)
cN, n (%)	
cN0	145 (52.7)
cN1a	58 (21.1)
cN1b	72 (26.2)
T, n (%)	
T1	201 (73.1)
T2	12 (4.4)
T3	50 (18.2)
T4	12 (4.4)

^a^Continuous variables are presented as means and standard deviations (SD); categorical variables are presented as n (%). *CLND* central-compartment lymph node dissection, *TT* total thyroidectomy, *LLND* lateral-compartment lymph node dissection

### Univariate analysis for level VII lymph node metastasis

In a univariate analysis, the relationship between level VII lymph node metastasis and clinicopathologic factors in the 275 patients was analyzed. Table 2 showed relationship between gender, age, poor differentiation, multifocality, papillary thyroid microcarinoma (PTMC), ultrasonography-positive lymph node metastasis (LNM), location of ultrasonography-positive LNM, gross lymph node metastasis, extrathyroidal extension, hashimoto thyroiditis (HT), location of primary carcinoma and level VII LNM. In our study, the rate of level VII LNM was significantly related with age, poor differentiation, multifocality, PTMC, ultrasonography-positive LNM, gross lymph node metastasis, extrathyroidal extension, location of primary carcinoma (*P* < 0.05). Patients with HT had a lower incidence of level VII lymph node metastasis compared with patients without HT (21.13% versus 31.37%), however, this was not of statistical significance (*P* = 0.128).

**Table 2:** Univariate analysis for level VII lymph node metastasis in 275 PTC patients

Variables^a^	Level VII lymph node metastasis		*P*-value
	Present	Absent	
Gender, n (%)	79	196	
Male	22 (34.92)	41 (65.08)	0.267
Female	57 (26.89)	155 (73.11)	
Age in years, mean (SD)	43.47 (13.32)	47.12 (11.33)	0.034
Poor differentiation, n (%)			0.001
Yes	7 (87.5)	1 (12.5)	
No	72 (26.97)	195 (73.03)	
Multifocality, n (%)			0.007
Yes	43 (37.72)	71 (62.28)	
No	36 (22.36)	125 (77.64)	
PTMC, n (%)			< 0.001
Yes	36 (20.57)	139 (79.43)	
No	43 (43.0)	57 (57.0)	
US (+) LNM, n (%)			< 0.001
Yes	43 (58.11)	31 (41.89)	
No	36 (17.91)	165 (82.09)	
Location of US (+) LNM, n (%)			0.155
Lateral Compartment	24 (50.0)	24 (50.0)	
Central Compartment	3 (75.0)	1 (25.0)	
Both	16 (72.73)	6 (27.27)	
Gross lymph node metastasis, n (%)			< 0.001
Yes	53 (41.09)	76 (58.91)	
No	26 (17.81)	120 (82.19)	
Extrathyroidal extension, n (%)			0.003
Yes	26 (45.61)	31 (54.39)	
No	53 (24.31)	165 (75.69)	
Hashimoto thyroiditis, n (%)			0.128
Yes	15 (21.13)	56 (78.87)	
No	64 (31.37)	140 (68.63)	
Location of primary carcinoma, n (%)			0.005
Upper third	14 (16.09)	73 (83.91)	
Middle third	29 (35.37)	53 (64.63)	
Lower third	36 (33.96)	70 (66.04)	

^a^Continuous variables are presented as means and standard deviations (SD); categorical variables are presented as n (%). *PTC* papillary thyroid carcinoma, *PTMC* papillary thyroid microcarcinoma, *US (+) LNM* ultrasonography-positive lymph node metastasis

### Multivariate logistic regression analysis for level VII lymph node metastasis

In the multivariate logistic regression analysis, location of primary carcinoma (*p* = 0.002), ultrasonography-positive LNM (OR = 11.788; 95% CI = 3.497–39.732; *p* < 0.001) and HT (OR = 0.422; 95% CI = 0.185–0.963; *p* = 0.04) were independently related with level VII LNM. Patients with ultrasonography-positive lymph nodes had a higher incidence of level VII LNM compared with patients with ultrasonography-negative lymph nodes (58.11% versus 17.91%). HT was a protective factor of level VII LNM. Patients with HT had a lower incidence of level VII LNM (21.13% versus 31.37%). The rates of level VII LNM for carcinoma locating in upper, middle and lower thirds in the lobe were 16.09, 35.37 and 33.96%, respectively. Table 3 illustrated differential risks based on the location the primary tumor resided in. Patients with primary tumor in the middle and lower third lobes had a greater probability of level VII LNM relative to patients with a primary tumor in the upper third (OR = 3.699; 95% CI = 1.546–8.849; *p* = 0.003 and OR = 4.278; 95% CI = 1.830–10.002; *p* = 0.001, respectively).

**Table 3:** Multivariate logistic regression analysis for level VII lymph node metastasis

Variables	***p***	OR	95%CI
Hashimoto Thyroiditis			
No		1 (reference)	
Yes	.040	0.422	0.185–0.963
Ultrasonography-positive LNM			
No		1 (reference)	
Yes	< 0.001	11.788	3.497–39.732
Location of primary tumor			
Upper third	.002	1 (reference)	
Middle third	.003	3.699	1.546–8.849
Lower third	.001	4.278	1.830–10.002

*LNM* lymph node metastasis

### Complications

There were no major or minor complications that occurred during the level VII lymph node dissection such as injury to major vessels, chyle leakage or pneumothorax. Hypoparathyroidism had the incidence rate with 23.64% (65/275), 1 of them had it permanently and needed oral calcium supplement. The incidence rate of recurrent laryngeal nerve injury was 1.82% (5/275).

## Discussion

Level VII lymph node metastasis from PTC is a well-recognized clinical phenomenon. The anatomical continuity of level VI and level VII has been long recognized, being first noted in 1956 by Crile [5], who described the lymphatic continuity of neck and the superior mediastinum, and again by Grebe in 1996 [6]. Metastasis of thyroid carcinoma to level VII lymph node may result from mechanistic downward retrograde route induced by an interruption of lymph circulation and direct extension into the superior mediastinum [7]. According to AJCC’s 8th edition, the N classification of level VII lymph node metastases is shifted from N1b to N1a. Moritani’s research showed level VII lymph node metastasis was not associated with worse disease-specific survival (DSS), however, it was associated with local recurrence [8]. It meant there was no difference between level VI and level VII in prognostic value.

We had the similar opinion with the level VII lymph node in accordance with AJCC’s 8th edition because of the anatomical continuity between level VI and level VII. However, the indication and prognostic value of prophylactic CLND were still inconsistent between different guidelines. The prominent scoring systems demonstrated that lymph node metastasis and the extent of cervical node dissection had no prognostic impact. However, some researchers showed that lymph node metastasis was an independent risk factor of DSS especially for high-risk patients [9, 10]. Since there was marginal evidence associating clinical significance of prophylactic CLND based on randomized studies, there had not been consensus on its application worldwide. Furthermore, the frequency of level VII LNM was relatively lower. Meanwhile, potential risk of complications, extra time of surgery (10–20 min), unfamiliar anatomical structure for surgeons and lack of prognostic data made the value of level VII dissection more controversial.

Besides, ultrasound evaluation had low sensitivity (50–70%) for detecting lymph node metastasis [11]. The level VII was particularly difficult to evaluate due to interference with bone structures of the chest wall. Failure to include level VII may leave significant macrometastatic nodal disease behind in both therapeutic and prophylactic dissections [12]. Though there are rare events, untreated subclinical lymph node metastasis could increase regional lymph node recurrence, increase the complexity of surgical treatment procedures and induce life-threatening problems due to their proximity to vital structures.

Above of all, we designed this study to determine the potential risk factors for Level VII LNM. Our study was the first study to present an analysis of predictive factors for Level VII LNM via video-assisted transcervical approach. Level VII LNM has been seen at variable incidence rates, ranging from 5.4 to 65% [7, 13–15], which is consistent with our study (28.73%). Previous studies suggested that many clinicopathological factors such as distant metastasis, greater total number of cervical LNM, tumor size, extrathyroidal extension, poorly differentiated carcinoma, and the age cut-off at 45 years were associated with Level VII LNM [7, 8, 13, 14]. However, most of them were based on the postoperative pathological information. According to our study, preoperative clinicopathological factors such as ultrasonography-positive LNM (*p* < 0.001) and location of the primary carcinoma (*p* = 0.002) were independent risk factors of level VII LNM whereas HT (*p* = 0.04) was independent protective factor of level VII LNM.

Patients with ultrasonography-positive lymph nodes were considered as high-risk patients. Study by Ito et al. demonstrated that PTC patients with clinically apparent (ultrasonography-positive) lateral-compartment lymph node have aggressive and progressive characteristics, and associated with worse outcomes based on DSS and DFS [16, 17]. Similarly, our study revealed that patients with ultrasonography-positive lymph node had higher incidences of level VII LNM (58.11% versus 17.91%). For PTC patients with ultrasonography-positive lymph nodes, level VII lymph node dissection could be considered. Further research regarding the impact of dormant positive lymph nodes in level VII on recurrence and survival is required to assess its significance.

The relationship between primary tumor location and the prevalence of lymph node metastasis had been studied for a long time [18, 19]. To our knowledge, this was the first study that associated the location of primary tumor to level VII lymph node metastasis in PTC patients. The relative incidence of level VII LNM for primary PTC in the upper, middle and lower thyroid regions was 16.09, 35.37 and 33.96%, respectively. Consistent with previous reports [18], we found that primary tumors located in the middle and lower third of the thyroid lobe conferred a high risk for level VII lymph node. This association is potentially based on the physiology of lymphatic drainage of the thyroid. The majority of lymphatic drainage follows the blood vessels that feed the gland. Ascending channels follow the superior thyroid artery and vein, draining the upper poles of the gland, while lymphatic drainage of the lower thyroid follows the inferior thyroid artery [20]. Since level VII lymph nodes were in anatomically continuous with level VI lymph nodes, it made sense that level VII LNM was associated with primary tumor location.

Both HT and PTC have a high prevalence worldwide, the relationship between PTC and HT was first described in 1955 by Dailey [21]. Since that time, numerous studies have examined their association, but it still remains a controversial issue. Some investigators have reported that PTC patients with HT tend to exhibit a lower frequency of lymph node metastases, less advanced disease, and better prognoses [22]. In contrast, other studies have demonstrated that when PTC is observed in conjunction with HT, it is more likely to be bilateral and multifocal, resulting in a higher frequency of lymph node metastases [23]. In our study, we noticed that HT played a protective role for level VII LNM, the frequency of level VII LNM in PTC patients with HT was less than that in the patients without HT (21.13 and 31.37% respectively). It was difficult to explain this conflicting phenomenon. One hypothesis indicated that elevated serum TSH rather than long-term HT was the risk factor for developing PTC [22]. Recent genome-wide association studies (GWAS) and hypothesis driven candidate gene approaches had determined that the FOXE1 genetic variant is downstream of the TSH-cAMP pathway, and is a suspected risk factor for follicular-cell-derived thyroid cancer [24].

## Conclusion

The Level VII lymph nodes are anatomically continuous with the Level VI lymph node. Based on the results of our study, we consider CLND should remain an integral strategy which includes the level VI and level VII lymph nodes. However, the frequency of level VII LNM was relatively lower. Meanwhile, potential risk of complications, extra time of surgery, unfamiliar anatomical structure and lack of prognostic data made VII dissection more controversial. On the basis of the surgery safety, transcervical level VII lymph node dissection could be considered for PTC patients with high risk factors such as ultrasonography-positive LN, tumor located in middle and lower thirds of the thyroid lobe and the patients without HT. The craniocaudal position, special self-developed thymus retractors and video-assisted approach facilitate the dissection. Furthermore, prognostic significance of level VII lymph node dissection should be evaluated through additional research and long-term surveillance.

## Data Availability

The datasets used and/or analyzed during the current study are available from the corresponding author on reasonable request.

## Abbreviations

AJCC: American Joint Committee on Cancer
ATA: American Thyroid Association
CI: Confidence interval
CLND: Central-compartment lymph node dissection
CT: Computed tomography
DSS: Disease-specific survival
ECG: Electrocardiogram
FNA: Fine-needle aspiration
FS: Frozen section
GWAS: Recent genome-wide association studies
HT: Hashimoto thyroiditis
LLND: Lateral-compartment lymph node dissection
LNM: Lymph node metastasis
MDT: Multiple disciplinary team
OR: Odds ratio
PTC: Papillary thyroid carcinoma
PTH: Parathyroid hormone
PTMC: Papillary thyroid microcarinoma
TSH: Thyroid stimulating hormone
TT: Total thyroidectomy
US: Ultrasound
